# Automated Home-Cage Testing as a Tool to Improve Reproducibility of Behavioral Research?

**DOI:** 10.3389/fnins.2020.00383

**Published:** 2020-04-24

**Authors:** Sophie Helene Richter

**Affiliations:** Department of Behavioural Biology, University of Münster, Münster, Germany

**Keywords:** reproducibility crisis, behavioral data, automation, experimenter effect, systematic heterogenization

## Introduction

Over the past few years, an intense discussion about the reproducibility of scientific findings in various life science disciplines arose (e.g., Baker, 2016; Scannell and Bosley, 2016; Voelkl and Würbel, 2016). Thereby, particular attention has been given to animal research, where irreproducibility prevalence rates have been estimated to range between 50 and 90% (Prinz et al., 2011; Collins and Tabak, 2014; Freedman et al., 2015). Highlighted under the umbrella term of the “reproducibility crisis,” mostly failures in the planning and conduct of animal experiments have been criticized to lead to invalid and hence irreproducible findings. To counteract these trends, animal scientists have repeatedly emphasized the need for a rethinking of current methodologies, mainly targeting aspects of the experimental design, but also of the reporting and publishing standards (see e.g., ARRIVE guidelines, Kilkenny et al., 2010; TOP guidelines, Nosek et al., 2016; du Sert et al., 2018; PREPARE guidelines, Smith et al., 2018).

However, as demonstrated by an already 20 years-old study, the use of thoroughly planned and well-reported protocols does not automatically lead to perfect reproducibility: In this study, three laboratories found remarkably different results when comparing the behavior of eight mouse strains in a battery of six conventionally carried out behavioral paradigms (Crabbe et al., 1999). It was hypothesized that “specific experimenters performing the testing were unique to each laboratory and could have influenced behavior of the mice. The experimenter in Edmonton, for example, was highly allergic to mice and performed all tests while wearing a respirator—a laboratory-specific (and uncontrolled) variable.” Following on from these thoughts, the importance of the experimenter as an uncontrollable background factor in the study of behavior moved into focus (e.g., Chesler et al., 2002a,b). Concomitantly, the use of automated test systems was promoted as a tool to reduce the experimenter's influence and to improve the accuracy and reproducibility of behavioral data (e.g., Spruijt and DeVisser, 2006).

Against this background, the present opinion paper aims at (1) briefly discussing the role of the experimenter in animal studies, (2) investigating the advantages and disadvantages of automated test systems, (3) exploring the potential of automation for improving reproducibility, and (4) proposing an alternative strategy for systematically integrating the experimenter as a controlled variable in the experimental design. In particular, I will argue that systematic variation of personnel rather than rigorous homogenization of experimental conditions might benefit the external validity, and hence the reproducibility of behavioral data.

## The Experimenter as an Uncontrollable Background Factor

As impressively illustrated by the above-mentioned multi-laboratory study, environmental conditions can exert a huge impact on behavioral traits. This has been particularly highlighted in the context of behavioral genetics, where experimental factors have been observed to interact greatly with trait-relevant genes. In order to identify and rank potential sources of such variability, Chesler et al. initially applied a computational approach to a huge archival data set on baseline thermal nociceptive sensitivity in mice (Chesler et al., 2002a,b). This way, they systematically identified several experimental factors that affected nociception, including, for example, season, cage density, time of day, testing order, or sex. Most interestingly, however, this analysis revealed that a factor even more important than the mouse genotype (i.e., the treatment under investigation) was the experimenter performing the test. Following on from this initial finding, subsequent studies provided further empirical evidence for the influence of the experimenter on the outcome of behavioral tests (e.g., van Driel and Talling, 2005; Lewejohann et al., 2006; López-Aumatell et al., 2011; Bohlen et al., 2014). With the aim of further disentangling what exactly constitutes the experimenter effect, some of these studies concentrated on specific characteristics of the personnel working with the animals. In particular, they could show that certain characteristics, such as the sex of the experimenter (Sorge et al., 2014) or the animals' familiarity with the personnel (van Driel and Talling, 2005) may play a crucial role.

Moreover, with respect to behavioral observations and direct experimenter-dependent assessments (e.g., counting “head-dips” on the elevated plus maze), it cannot be ruled out that the human observer may evaluate observations inconsequently and that definitions of behavior in, for example, ethograms are interpreted in various ways. Training deficits as well as a lack of inter-observer reliability can thus be regarded as additional sources of the experimenter-induced variation (Spruijt and DeVisser, 2006; Bohlen et al., 2014). Furthermore, only short habituation periods may promote differential reactions of individual animals toward the observer. Irrespective of the precise features that account for the described experimenter effects, however, the research community has widely agreed upon the importance of this factor as an uncontrollable background factor in behavioral research.

## The Use of Automated Test Systems in Behavioral Studies

To overcome this issue, voices became loud during the last years to increase the usage of automated and experimenter-free testing environments, particularly in behavioral studies. Thereby, automation is not only considered beneficial to reduce or even prevent the confounding impact of the “human element”, but also to decrease the time-consuming efforts of human observers. Looking at the literature, two major research lines have been pursued to implement this idea further: (1) Automation of recording and test approaches, and (2) development of automated home cage phenotyping, or alternatively, test systems that are attached to home cages and can be entered on a voluntary basis. Whereas, the former involves testing the animal outside of the home cage and thus still requires handling by an experimenter (e.g., Horner et al., 2013), the latter enables a completely new route for monitoring behavior over long periods of time within the familiar environment and without any need for human intervention (e.g., Jhuang et al., 2010). With regard to automated test systems used outside of the home cage, typical examples are touchscreen chambers, mainly used for the assessment of higher cognitive functions in rodents (e.g. Bussey et al., 2008, 2012; Krakenberg et al., 2019), Skinner boxes (e.g. Rygula et al., 2012), or more specifically targeted technologies, such as the automated maze task (Pioli et al., 2014), the automated open field test (Leroy et al., 2009), or the automated social approach task (Yang et al., 2011). Likewise, systems, such as the IntelliCage (e.g., Vannoni et al., 2014), the PhenoCube (e.g., Balci et al., 2013), or the PhenoTyper (e.g., De Visser et al., 2006) have been developed to track the behavior within the familiar home cage. Potential advantages of such automated compared to manual assessments include the continuous monitoring, particularly during the dark phase when mice are most active, the observation in a familiar and thus less stressful environment, and the examination of combinations of behaviors rather than single behaviors (Steele et al., 2007). The latter point has been particularly highlighted as being crucial for the behavioral characterization of rodent disease models, as signs of ill health, pain, and distress tend to be very subtle in these animals (Weary et al., 2009). Furthermore, letting animals self-pace their task progression from a home-cage has been shown to speed up learning and to increase test efficiency in complex tasks (e.g., 5-choice serial reaction time task, Remmelink et al., 2017). Lastly, the use of automated technologies allows animals to maintain some control over which resources they would like to interact with, a key advantage in terms of animal welfare (Spruijt and DeVisser, 2006). At the same time, automation may come with certain challenges: For example, many automated test systems are not yet adapted to group housing and thus may require single housing of the study subjects, at least during the observation phases. This in turn may critically impair the welfare of these individuals, and undermines the goal of refining housing conditions for social animals according to their needs (Richardson, 2012; but see also Bains et al., 2018). Likewise, even the best automation does not prevent the individual from being handled for animal care reasons, probably potentiating the stress experienced during these rare events. Although the increasing implementation of automated systems in behavioral studies thus brings about a number of advantages, there is still room and need for further improvement.

## Automation and Reproducibility of Behavioral Data

In light of the hotly discussed reproducibility crisis, automation is especially promoted as one potential way out of the problem. In particular, it has been argued that a computer algorithm, once programmed, and trained is consistent and unbiased and may thus reduce unwanted variation and hence contribute to improved comparability and reproducibility across studies and laboratories (Spruijt and DeVisser, 2006; Spruijt et al., 2014). In line with these arguments, a behavioral characterization of C57BL/6 and DBA/2 mice in the PhenoTyper indeed revealed highly consistent strain differences in circadian rhythms across two laboratories (Robinson et al., 2018). Likewise, automated home-cage testing in IntelliCages was found to provide consistent behavioral and learning differences between three mouse strains across four laboratories, i.e., no significant laboratory-by-strain interactions could be detected (Krackow et al., 2010). Furthermore, comparing this system with conventional testing of mice in the open field and the water maze tests yielded more reliable results in the IntelliCages, even though the conventional tests were standardized strictly (Lipp et al., 2005). All of these studies indeed hint toward improved reproducibility through automation, suggesting that the absence of human interference during behavioral testing is a prominent advantage. However, systematic investigations on this topic are still scarce, in particular when it comes to comparisons to conventional approaches. Furthermore, significant behavioral variation has also been found to occur among genetically identical individuals that lived in the same “human-free” environment (Freund et al., 2013), indicating that the link between absence of human interference, reduced variation, and better reproducibility is not straightforward. As an alternative to improving reproducibility through automation, one may thus also think about turning the experimenter effect into something “advantageous” by systematically considering this factor in the experimental design. So, what exactly is meant by this?

## Discussion—Alternative Strategies to Improve Reproducibility

Following the above-presented logic, automated test systems reduce the influence of the experimenter, and may therefore be characterized by a higher degree of within-experiment standardization. Typically, it is argued that such increased standardization reduces variation, thereby improves the test sensitivity, and hence allows for detecting statistically significant effects with a lower number of animals (e.g., Richardson, 2012). At the same time, however, it has been pointed out that rigorous standardization limits the inference to the specific experimental conditions, thereby boosting any laboratory-specific deviations. Increasing the test sensitivity through rigorous standardization therefore comes at the cost of obtaining idiosyncratic results of limited external validity [referred to as “standardization fallacy” by Würbel (2000, 2002)]. Instead, the use of more heterogeneous samples has been suggested to make study populations more representative and the results more “meaningful” (Richter et al., 2009, 2010, 2011; Voelkl and Würbel, 2016; Richter, 2017; Milcu et al., 2018; Voelkl et al., 2018; Bodden et al., 2019). According to this idea, the introduction of variation on a systematic and controlled basis (referred to as “systematic heterogenization” by e.g., Richter et al., 2009, 2010; Richter, 2017) predicts increased external validity and hence improved reproducibility.

**Figure 1:**
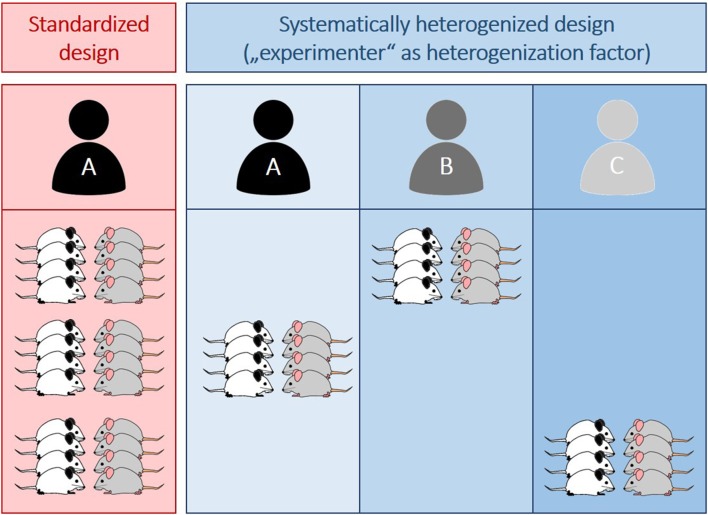
Illustration of a conventionally standardized (red) and a systematically heterogenized experimental design (blue). Whereas in the standardized design all animals (*n* = 12 per group) are tested by one experimenter (A), three different experimenters (A–C) are involved in the systematically heterogenized design. Importantly, animals are assigned to the experimenter in a random, but balanced way with each person testing the same amount of animals per group (*n* = 4 per group and experimenter). Different colors of mice indicate different groups (e.g., different pharmacological treatments or genotypes).

As outlined above, increased automation may entail similar risks as it excludes one factor (i.e., the experimenter) known to induce or explain a lot of variation in behavioral studies. This way, conditions within experiments are more stringently homogenized, increasing the risk for obtaining spurious findings. Instead of trying to eliminate this variation, one may therefore think about systematically including it to improve the overall robustness of the data. Building on previous heterogenization studies (e.g., Richter et al., 2010; Bodden et al., 2019), this would simply mean to vary the within-experiment conditions by systematically involving more than just one experimenter. More precisely, instead of including one experimenter, who is responsible for testing all animals of one experiment (“conventional standardized design”, Figure 1), animals could for example be split in three equal groups (balanced for treatment, see also Bohlen et al., 2014), each tested by a different person (“systematically heterogenized design”, Figure 1).

From a practical perspective, such an approach may be associated with certain challenges, especially for small research groups with limited resources. For bigger research organizations or large-scale testing units, however, the organizational efforts might increase only marginally. Balancing overall costs and benefits, such an experimenter-heterogenization may still represent an effective and easy-to-handle way to maximize the informative value of each single experiment (see Richter, 2017). Thus, rather than eliminating the uncontrollable factor “experimenter”, it could be turned into a controllable one that—systematically considered—may in fact benefit the outcome of behavioral studies.

## Author Contributions

The author confirms being the sole contributor of this work and has approved it for publication.

## Conflict of Interest

The author declares that the research was conducted in the absence of any commercial or financial relationships that could be construed as a potential conflict of interest. The handling editor declared a past co-authorship with the author SR.
